# Antibiotic Susceptibility Testing of the Gram-Negative Bacteria Based on Flow Cytometry

**DOI:** 10.3389/fmicb.2016.01121

**Published:** 2016-07-26

**Authors:** Claude Saint-Ruf, Steve Crussard, Christine Franceschi, Sylvain Orenga, Jasmine Ouattara, Mahendrasingh Ramjeet, Jérémy Surre, Ivan Matic

**Affiliations:** ^1^Institut National de la Santé et de la Recherche Médicale, Sorbonne Paris Cité, Faculté de Médecine Paris Descartes, Université Paris DescartesParis, France; ^2^Microbiology Unit, R&D Microbiology, BioMérieux SALa Balme Les Grottes, France

**Keywords:** flow cytometry, antibiotic susceptibility testing, protein carbonylation, fluorescent hydrazide, bacteria

## Abstract

Rapidly treating infections with adequate antibiotics is of major importance. This requires a fast and accurate determination of the antibiotic susceptibility of bacterial pathogens. The most frequently used methods are slow because they are based on the measurement of growth inhibition. Faster methods, such as PCR-based detection of determinants of antibiotic resistance, do not always provide relevant information on susceptibility, particularly that which is not genetically based. Consequently, new methods, such as the detection of changes in bacterial physiology caused by antibiotics using flow cytometry and fluorescent viability markers, are being explored. In this study, we assessed whether Alexa Fluor® 633 Hydrazide (AFH), which targets carbonyl groups, can be used for antibiotic susceptibility testing. Carbonylation of cellular macromolecules, which increases in antibiotic-treated cells, is a particularly appropriate to assess for this purpose because it is irreversible. We tested the susceptibility of clinical isolates of Gram-negative bacteria, *Escherichia coli* and *Pseudomonas aeruginosa*, to antibiotics from the three classes: β-lactams, aminoglycosides, and fluoroquinolones. In addition to AFH, we used TO-PRO®-3, which enters cells with damaged membranes and binds to DNA, and DiBAC4 (3), which enters cells with depolarized membranes. We also monitored antibiotic-induced morphological alterations of bacterial cells by analyzing light scattering signals. Although all tested dyes and light scattering signals allowed for the detection of antibiotic-sensitive cells, AFH proved to be the most suitable for the fast and reliable detection of antibiotic susceptibility.

## Introduction

Treating bacterial infections with an appropriate antibiotic is of the utmost importance. A systematic review and meta-analysis revealed that the percentage of in-hospital inappropriate empiric antibiotic use ranged from 14 to 79% (Marquet et al., 2015). Inappropriate antibiotic treatments considerably increase morbidity and mortality rates, even if the patient receives an appropriate antibiotic as a second line therapy (Davey and Marwick, 2008; Marquet et al., 2015). Inappropriate antibiotic treatments needlessly expose patients to unwanted side effects and contribute to the increase in the appearance and spread of multidrug resistance (Kohanski et al., 2010). Consequently, inappropriate antibiotic therapies are significantly contributing to an increase in healthcare costs that are already high. The inappropriate utilization of antibiotics also decreases their effective lifespan, which is of major concern because of a shortage in the discovery of new antibiotics mostly due to the lack of interest of the pharmaceutical industry (Fair and Tor, 2014). Consequently, there is a lack of promising candidate compounds that passed clinical trials.

Fast and accurate antibiotic susceptibility tests can significantly reduce mortality rates and reduce financial costs (Barenfanger et al., 1999). Furthermore, the necessity of rapidly prescribing an initial empirical antimicrobial treatment while waiting for the susceptibility test results from time—consuming standard methods frequently leads to inappropriate treatments (Ibrahim et al., 2000). The standard methods of antibiotic susceptibility testing (AST) are based on measuring the inhibition of bacterial growth. Consequently, the average times required to obtain a result are ~18–24 h for the most rapid systems. Standard techniques include the broth microdilution reference method, as well as manual and automated alternative methods, such as Etest®, VITEK 2 (bioMérieux, Marcy l'Etoile, France) and disk diffusion (Eigner et al., 2005; Courvalin et al., 2009). Most of these methods also measure the minimum concentration of an antibiotic that is able to inhibit the growth of a bacterial strain. This value, referred to as the minimum inhibitory concentration (MIC), was first defined in the mid-1940s (Wheat, 2001).

In the last two decades, faster AST methods, such as DNA-based tests (Barken et al., 2007) and mass spectrometry-based methods (Opota et al., 2015; Trip et al., 2015), have been developed. However, they do not always provide relevant information on antibiotic susceptibility. For example, DNA-based tests rely on the knowledge of the resistance mutations and genes, which is far from being exhaustive. In addition, bacteria lacking resistance mutations and genes may still be able to tolerate and survive antibiotic treatments by utilizing many other mechanisms, some of which are non-genetic (Javid et al., 2014; Sanchez-Romero and Casadesus, 2014). This obstacle in testing could be circumvented by detecting the changes in bacterial physiology caused by antibiotics using flow cytometry (FC) and fluorescent viability markers, which has been demonstrated by numerous studies (Gant et al., 1993; Walberg et al., 1997; Gauthier et al., 2002; Ambriz-Avina et al., 2014). A major advantage of this methodological approach is that some of the physiological changes caused by antibiotics occur long before growth inhibition is observable (1 h vs. 1 or 2 days).

Despite its great potential, FC-based AST (FC-AST) methods are not yet used for routine clinical applications. The major difficulty of FC-AST methods is the choice of appropriate phenotypic markers. For example, changes in membrane integrity or a loss of membrane potential can be induced even by sublethal doses of antibiotics but can be reversible and highly variable (Huang et al., 2015). The best markers are those associated with the irreversible impairment of cell viability. The carbonylation of proteins is one such marker because it is an irreversible process induced by various lethal stressors (Burcham and Fontaine, 2001; England et al., 2004), including antibiotic treatment (Kolodkin-Gal et al., 2008; Tkachenko et al., 2012; Belenky et al., 2015). Recently, Belenky et al. showed that antibiotic-treated cells characteristically exhibited high levels of protein carbonylation and a panel of cytotoxic changes that most probably resulted from oxidative stress (Kohanski et al., 2010; Belenky et al., 2015). Increased reactive oxygen species levels result also in carbonylation of other cellular macromolecules like lipids and nucleic acids (Vemula et al., 2015). Importantly, carbonylation continues even after cell death (Dalle-Donne et al., 2006). Therefore, this process is potentially an effective marker for use in FC-AST applications. Carbonylated molecules react with fluorescent hydrazides (Ahn et al., 1987), which only enter cells with compromised membranes. Fluorescent hydrazide-stained carbonylated molecules can be detected using FC. In a previous study, we demonstrated that the staining of bacterial cells with Alexa Fluor® 633 Hydrazide (AFH) is an indication that they are not able to recover from lethal stress (Saint-Ruf et al., 2010).

In this study, we evaluated the potential of the AFH for use in FC-AST using clinical isolates of Gram-negative bacteria, *Escherichia coli* and *Pseudomonas aeruginosa*, as model organisms. Bacteria were treated with drugs from three antibiotic classes: β-lactams, aminoglycosides, and fluoroquinolones. In addition to the level of carbonylation, we monitored the following parameters, which have also previously been reported to vary in antibiotic-treated bacteria (Gant et al., 1993; Gauthier et al., 2002; Ambriz-Avina et al., 2014): (i) the loss of membrane integrity using TO-PRO®-3 dye (Kerstens et al., 2014), which enters cells with compromised membranes and binds to nucleic acids; (ii) the loss of membrane potential using DiBAC4(3) dye, which enters cells with depolarized membranes (Joux and Lebaron, 2000); and (iii) the alterations of the light scattering parameters, forward scatter (FSC), and side scatter (SSC), which indicate the size and protein content of the cells, respectively. We also evaluated the relevance of FC analysis for deducing the MIC of β-lactams for clinical isolates of *E. coli* and *P. aeruginosa*.

## Materials and methods

### Bacterial strains and dyes

All bacterial strains were obtained from the bioMérieux collection Table 1. The following dyes were purchased from Invitrogen (Carlsbad, CA, USA): Alexa Fluor 633 Hydrazide (AFH) (absorption 630 nm, emission 645 nm), TO-PRO®-3 (absorption 642 nm, emission 661 nm), and DiBAC4(3) (absorption 540 nm, emission 590 nm).

**Table 1:** **Bacterial strains used in this study**.

**STRAINS**	**ANTIBIOTICS**	**MIC mg/L**	**INTERPRETATION**
***E. coli***			
ATCC 25 922	Ampicillin	4	S
	Cefoxitin	4	S
	Imipenem	0.125	S
	Gentamicin	0.5	S
	Ciprofloxacin	≤0.0039	S
9203096	Ampicillin	4	S
8812112	Ampicillin	64	R
1110054	Cefoxitin	32	R
1211021	Imipenem	16	R
9607098	Gentamicin	32	R
9210041	Ciprofloxacin	8	R
***P. aeruginosa***			
9203182	Imipenem	1	S
9405057		2	S
9504020		2	S
9412033		4	S
9508040		8	R
0206035		16	R
9207006		16	R
1011314		16	R
9207013		32	R

*Escherichia coli 9203096 and Pseudomonas aeruginosa 9203182 strains correspond to the CDC 2039 and CDC 2087 strains, respectively. These two strains are available at the Centers for Disease Control, Atlanta, Georgia, USA. Other strains can be obtained upon request from bioMérieux*.

### MIC determination

The lowest concentration of an antibiotic that completely inhibited visible growth as detected by the unaided eye was recorded as the MIC. MICs were determined using the broth microdilution reference method as described by the Clinical and Laboratory Standards Institute (CLSI) (Patel et al., 2014). MICs were determined by examining the growth of each strain for at least three replicates. If two of the three results were the same, that MIC was used as the composite (“voted”) result. If the three results differed, the middle result was used. CLSI breakpoints were applied to determine category interpretations, i.e., susceptible or resistant (Patel et al., 2014).

### Growth conditions

The media utilized were lysogeny broth (LB) broth and LB agar (Difco, Lawrence, KS, USA). Bacteria from a frozen glycerol stock were inoculated in 10 ml of LB medium in 50 ml centrifuge polypropylene tubes (Sardtedt, Numbrecht, Germany). Bacteria were grown in an agitated, liquid LB medium at 37°C overnight. This culture was diluted 10^4^-fold in LB medium and grown again overnight to obtain a starter culture for the antibiotic treatments.

### Antibiotic treatment procedure

Fresh LB medium was inoculated with bacteria from the starter culture to give an initial inoculum of 0.01 OD_600_, which was allowed to continue to grow for 1 h, and the culture was then divided into equal volumes and placed into tubes in the presence or absence of an antibiotic. The following antibiotics were used: ampicillin (Totapem) (Unipex, Paris, France), cefoxitin (Mefoxin) (Sigma-Aldrich, Saint-Louis, MO, USA), imipenem (Tienam) (bioMérieux Inc Saint-Louis, MO, USA), gentamicin (Gentalline) (Unipex, Paris, France), and ciprofloxacin hydrochloride (Ciflox) (MP Biomedical, Santa Ana, CA, USA). Each sample was incubated for at least 4 h at 37°C. Each hour, aliquots of cells were taken, stained with the dyes described below, and analyzed via FC. The number of colony forming units (CFUs) were also counted.

### CFU analysis

To establish the number of CFUs per ml of liquid culture, aliquots of cells, or of the diluted cells, were spread on LB agar plates (agar 15 g/liter) and incubated overnight at 37°C, and the number of CFUs was then counted.

### Staining procedure

Based on the concentration of cells, the bacterial culture was diluted to obtain 10^7^ cells per ml in filtered (pore size, 0.22 μm) phosphate-buffered saline (PBS; Dulbecco Bio-Rad, California UK) supplemented with one of the dyes. Alternatively, cells were harvested from culture broth via centrifugation at 8000 × *g* for 3 min and resuspended at a concentration of 10^7^ cells per ml in PBS supplemented with one of the dyes. Staining is not density dependent. Even small number of cells can be successfully stained. AFH, TO-PRO®-3 and DiBAC4(3) were used at concentrations of 2 μg/ml, 0.5 μM, and 10 μg/ml in PBS, respectively. Cells were incubated at room temperature for 15 min in the dark. Samples stained with AFH were washed in PBS before FC analysis, whereas samples stained with TO-PRO®-3 or DiBAC4(3) were immediately analyzed via FC.

### Flow cytometry

We used a BD Accuri C6 FACS system (Becton Dickinson Biosciences, Franklin Lakes, NJ) to analyze and count the bacterial cells. Blue laser light (488 nm) was used to analyze DiBAC4(3) (filters, 530/30 nm). The 640 nm diode was used to analyze Alexa Fluor 633 hydrazide and TO-PRO®-3 (filters, 675/25 nm). Samples were run at the rate of 2000 events/s; 50,000 events were collected. Data acquisition and analysis were performed with BD Accuri C6 and FlowJo software (FLOWJO LLC, Ashland, Oregon, USA).

### ImageStream cytometry

All samples were acquired on an ImageStreamX MarkII system (Merck Millipore, Darmstadt, Germany) using a 488 nm laser (BP 505–560) and a 642 nm laser (BP 640–745). A total of 5000 events were collected for each sample. IDEAS software (Amnis Corporation, Seattle, WA) was used to analyze the measurements.

### Measurement of the FSC/SSC areas

The FSC/SSC area for each experiment was estimated from pseudocolor density-plots FSC/SSC. The density-plots were saved as a JPEG files and analyzed with ImageJ 1.48 v software. The perimeter of the cloud of dots was delimited using the “polygon selection” tool. Isolated dots outside the cloud were not included in this perimeter (Figure S7), and the defined area was measured.

## Results

### Detection of the susceptibility of *Escherichia coli* strains to different antibiotics

We first analyzed the susceptibility of *E. coli* strains to different antibiotics. These included β-lactam antibiotics (ampicillin, cefoxitin, and imipenem), which inhibit cell wall biosynthesis; an aminoglycoside (gentamicin), which inhibits protein synthesis by binding to the 30S ribosomal subunit; and a fluoroquinolone (ciprofloxacin), which binds to the DNA-topoisomerase complexes, resulting in DNA breaks and DNA replication fork arrest (Kohanski et al., 2010). The MICs of all antibiotics for each bacterial strain were determined using standard microdilution AST methods (Table 1). For each experiment, log-phase cultures of a sensitive strain and of a strain resistant to a given antibiotic (Table 1) were or were not treated with the given antibiotic and were then analyzed in a 4-h time course study. The strains sensitive to a given β-lactam were treated with the MIC of these antibiotics. The gentamicin-sensitive strain and the ciprofloxacin-sensitive strain were treated with gentamicin and ciprofloxacin at 8 × MIC, respectively. The resistant strains received the same treatment as the sensitive strains.

### Measuring the bacterial cell number and CFU counts

To evaluate the ability of the antibiotics to kill *E. coli* and inhibit growth, we measured the number of bacterial cells via FC, which scores live and death cells, and the number of CFUs during 4 h (Figure S1A). The number of cells of an untreated sensitive strain and of an untreated and treated resistant strain increased in a similar manner over time. The time-dependent increase in the number of the cells in the treated, sensitive population was impaired when compared to the change in the untreated control. The number of sensitive cells in the samples treated with ciprofloxacin did not increase and was relatively stable over time. The median number of sensitive cells increased 1.3-fold after treatment with ampicillin and three-fold after treatments with cefoxitin and imipenem during first hour of treatment, after which, it slightly decreased. The number of sensitive cells in samples treated with gentamicin increased ~10-fold, which amounts to approximately three cell doublings, during first hour of incubation, after which, it remained stable. CFU counts for the treated, sensitive strain continuously decreased, while CFU counts for the untreated, sensitive strain and of untreated and treated, resistant strain increased during the 4 h of incubation for all antibiotics (Figures S1A,B). Therefore, it can be concluded that the fraction of cells unable to grow and form colonies on LB plates increased within the sensitive populations after treatment with the antibiotics. Even cells that succeeded in dividing during the first hour of treatment were unable to give rise to colonies.

### Light scattering profiles FSC and SSC

**Figure 1:**
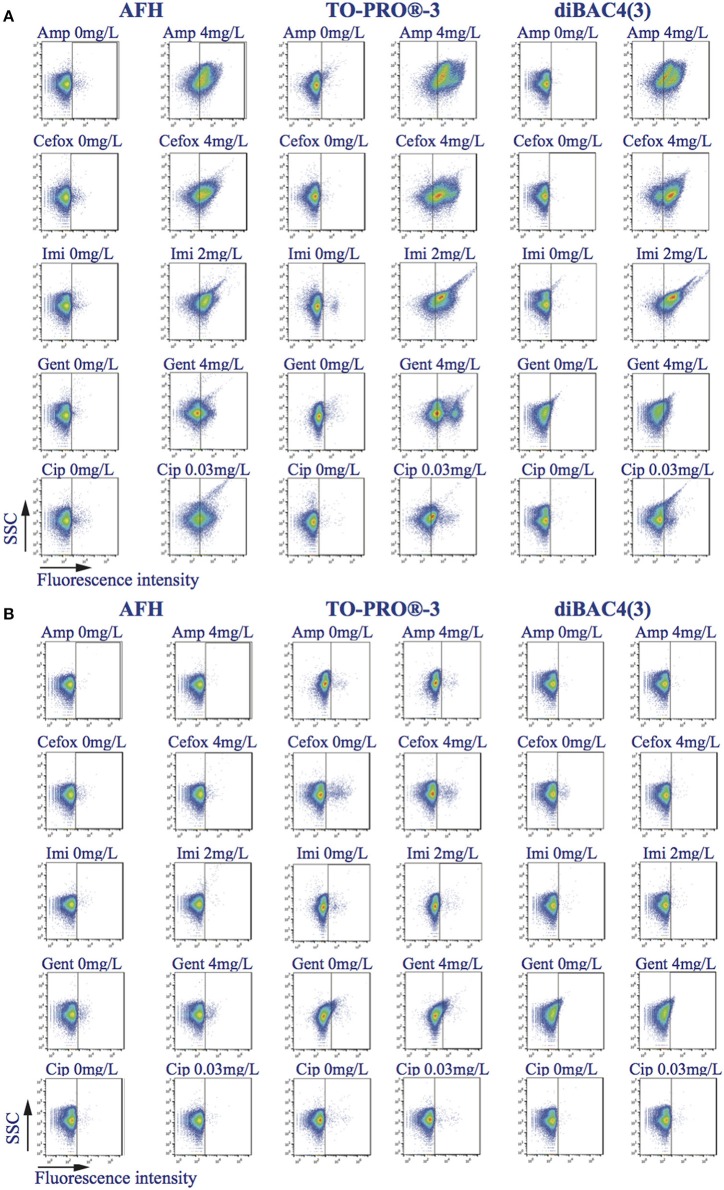
**Effects of antibiotics on the light scatter SSC and fluorescence intensity distribution**. Density-plots SSC vs. fluorescence intensity of bacterial samples treated, or not, with ampicillin, cefoxitin, imipenem, gentamicin, and ciprofloxacin, during 2 h and stained with AFH, TO-PRO®-3, and DiBAC4(3). The vertical bars in the density-plots of the untreated samples and the density-plots of the treated samples are at the same place. **(A)** *E. coli* ATCC25922 strain, which is sensitive to all the studied antibiotics. **(B)** *E. coli* strains resistant to the one of the tested antibiotics.

After incubation with ampicillin, the light scattering profile of the treated, sensitive *E. coli* strain became dispersed due to the appearance of cells of different shapes and sizes relative to the profile of the untreated control (Figure 1, Figures S2, S4). After 1 h of incubation, cells with sizes ranging from 4 to 166 μm, along with numerous filamentous cells, were observable in images acquired with the ImagestreamX MarkII FC system. After 2 h, a population of cells smaller than those of the untreated sample appeared alongside the population of larger cells (Figure S4B). These observations suggest that many filamentous cells or large cells may have undergone lysis, divided and given rise to smaller cells or shrunk. As a measure of the variation of the light scattering profiles, we calculated the FSC (or SSC) ratio, which is equal to the mean value of the FSC (or SSC) intensity of the treated cells divided by the mean value of the FSC (or SSC) intensity of the untreated cells. After 2 h of treatment with ampicillin, imipenem, gentamicin or ciprofloxacin, the FSC ratios of the sensitive strain were significantly (*t*-test, *p* < 0.05) higher (2 to 10-fold depending on the antibiotic) than the FSC ratios of the resistant strains. Only the FSC ratio of the sensitive strain treated with cefoxitin was not significantly different than the FSC ratio of the resistant strain. The SSC ratios of the sensitive strain were significantly (*t*-test, *p* < 0.05) higher (two and six-fold) than those of the resistant strains after only 2 or 3 h of treatment with ampicillin and ciprofloxacin.

**Figure 2:**
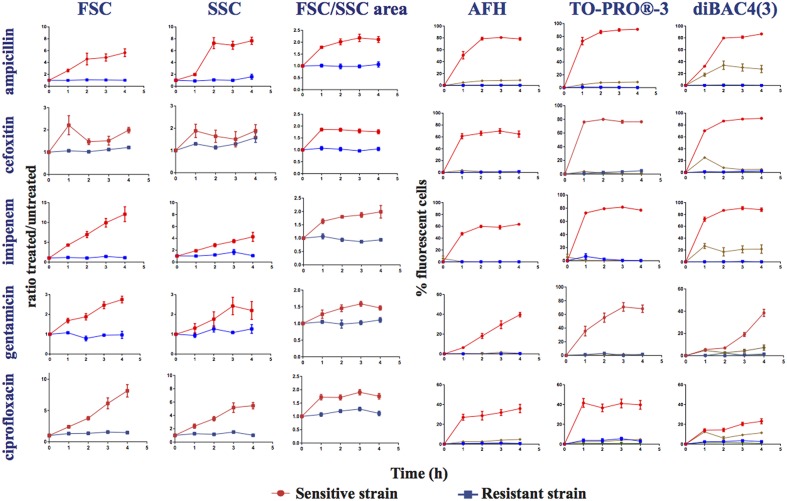
**Monitoring alterations of the *E. coli* strains treated with different antibiotics via flow cytometry**. Time course study of sensitive (ATCC25922 strain sensitive to all the studied antibiotics) and resistant strains treated, or not, with ampicillin, cefoxitin, imipenem, gentamicin, and ciprofloxacin. This figure shows the ratios of the mean FSC intensity of the antibiotic treated samples/the mean FSC intensity of the untreated samples; the ratios of the mean SSC intensity of the treated samples/the mean SSC intensity of the untreated samples; the ratios of the area of the density plots for FSC/SSC (FSC/SSC area) of the antibiotic-treated samples/the FSC/SSC area of the untreated samples; and the percentage of fluorescent cells stained with AFH, TO-PRO®-3 and DiBAC4(3) of the antibiotic-treated samples, all relative to incubation time. The percentage of fluorescent cells in treated samples was determined using the fluorescence of the untreated samples as a baseline. 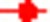, sensitive antibiotic-treated strain, 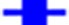, resistant antibiotic-treated strain, 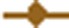, autofluorescence of the sensitive antibiotic-treated strain, 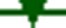, autofluorescence of the antibiotic-treated resistant strain (Ex 640 nm, Em 675 nm ± 25 nm for AFH and TO-PRO®-3; Ex 488 nm, Em 530 nm ± 30 nm for DiBAC4(3)). Each value represents the mean value obtained from four independent experiments. The error bars correspond to the standard error of the mean.

Because the changes in the light scattering profile caused by the antibiotics within the treated, sensitive populations occur in two opposite directions, i.e., some cells were smaller and some larger than the untreated cells, an analysis of the FSC and SSC mean intensity values can lead to the underestimation of these changes. Therefore, for a better estimation of the light scattering variations, we also calculated the area of scattering on density dot-plots for FSC/SSC for each experiment. Afterward, we calculated the ratio of the area of scattering of the treated sample vs. that of the corresponding untreated sample. Based on this calculation, we observed a significant difference in the light scattering of the cells treated with all tested antibiotics after 1 or 2 h (*t*-test, *p* = 0.03). These results indicate that measurements of the dispersion of the light-scattering profiles of the cells after antibiotic treatment allow for the estimation of the susceptibility of a strain to an antibiotic (Figure 2).

### Autofluorescence

We observed that the antibiotic treatments of the sensitive *E. coli* strain induced a weak far-red cellular autofluorescence. Less than 5% of the treated cells had a higher autofluorescence than the autofluorescence of the untreated cells as detected with the FC parameters used to measure the fluorescence of AFH and TO-PRO®-3 (excitation at 633 nm with bandpass filters of 660/20 nm; Figure 2). In contrast, the antibiotics induced a substantial green autofluorescence (excitation at 488 nm with bandpass filters of 530/30) in the sensitive cells. 25 to 35% of the sensitive cells treated with the three β-lactams and 5 to 15% of the sensitive cells treated with gentamicin and ciprofloxacin had autofluorescence intensities 10-fold higher than the untreated cells (Figure 2). Treating resistant bacterial strains with antibiotics did not increase cellular autofluorescence. While the antibiotic-induced autofluorescence was low to interfere with the measurements of fluorescence the antibiotic-treated cells stained with AFH and TO-PRO®-3, it could interfere with the measurement of the green fluorescence of antibiotic-treated cells stained with DiBAC4(3).

### Antibiotic-induced changes revealed by staining with AFH, TO-PRO®-3, and DIBAC4

We stained treated and untreated *E. coli* cells with the dyes AFH, TO-PRO®-3, and DiBAC4(3), and compared the changes in the distributions of fluorescent signal intensity using FC (Figures 1, 2, Figures S2, S3). First, we analyzed the mean fluorescence intensity (MFI) of cell populations treated or untreated with the antibiotics during 5 h and stained with the AFH and TO-PRO®-3 (Figure S3). We found a increase of the MFI of the sensitive cell population treated with the antibiotics for both dyes. An increase of the MFI for AFH stained cells correlated with increasing mortality (CFU). However, this was true for TO-PRO®-3 only during first 2 h of incubation with antibiotic, because of the MFI decrease after 2 h of incubation.

Second, we analyzed the percentage of stained cells to evaluate number of cells impacted by the antibiotic. The percentage of cells that were fluorescent due to the antibiotic treatment were calculated using the fluorescence of untreated stained cells as a baseline fluorescence. For each antibiotic, the percentage of cells from the resistant strains stained with the three dyes did not exceed 3%.

A substantial increase in the percentage of fluorescent cells stained with AFH was observed for the sensitive strain after 1 h of treatment with all antibiotics (Figure 2). After 2 h of incubation with ampicillin, cefoxitin, and imipenem, the percentage of fluorescent cells reached plateaus of 80 and 60%, respectively. This is in accordance with observations that this strain survives cefoxitin and imipenem treatment better than it does ampicillin treatment (Figure S1). A similar plateau, at ~30%, was observed for the cell populations treated with ciprofloxacin, while percentage of fluorescent cells treated with gentamicin continuously increased up to 40%. The different kinetics of AFH staining associated with the different antibiotics probably reflects different kinetics of membrane damage and carbonylation of macromolecules, which are both required to obtain a fluorescent signal using this dye.

As with AFH, a substantial increase of the percentage of cells stained with TO-PRO®-3 was observed for the sensitive strain after 1 h of treatment with all antibiotics (Figure 2). The patterns of staining with TO-PRO®-3 after treatment with all antibiotics were similar to those obtained with the AFH treatment except that the percentage of stained cells was 10 to 20% higher in the β-lactam-treated samples.

The results obtained with DiBAC4(3) staining were different than the results from the two other dyes. A substantial increase in the percentage of stained cells was observed for the sensitive strain after 1 h of incubation with β-lactams but not with gentamicin or ciprofloxacin (Figure 2). Plateaus of 80% fluorescent cells were observed after 2 h of incubation with the 3 β-lactams. For the gentamicin treatment, 20 and 40% of the cells were stained after 3 and 4 h, respectively. After 4 h of incubation with ciprofloxacin, only 25% of cells were stained. Taken together, our results indicate that AFH and TO-PRO®-3 are better markers than DiBAC4(3) for use in FC-AST for a wide range of antibiotics.

### Correlation between the MIC of ampicillin and flow cytometry data

**Figure 3:**
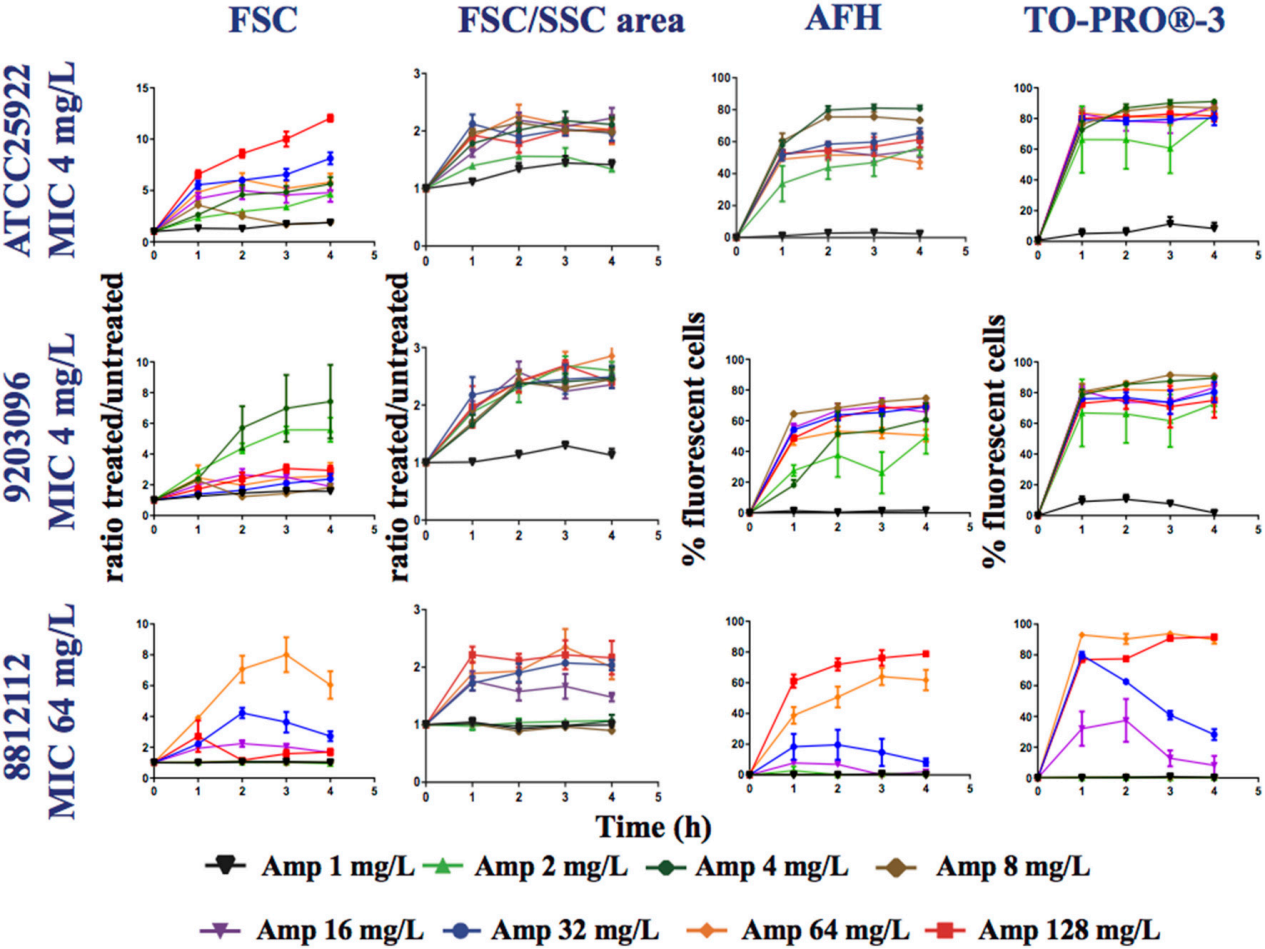
**Monitoring alterations of the *E. coli* strains treated with a range of ampicillin concentrations via flow cytometry**. The *E. coli* 7705035 (MIC = 4 mg/ml), 9203096 (MIC = 4 mg/ml), and 8812112 (MIC = 64 mg/ml) strains were treated with a range of ampicillin concentrations (0, 1, 2, 4, 8, 16, 32, and 64 mg/L). This figure shows the ratios of the mean FSC intensity of the ampicillin treated samples/the mean FSC intensity of the untreated samples, the ratios of the FSC/SSC area of the ampicillin treated samples/the FSC/SSC area of the untreated samples and the percentage of fluorescent cells stained with AFH and TO-PRO®-3, all relative to incubation time. Each value represents the mean value obtained from four independent experiments. The error bars correspond to the standard error of the mean.

To evaluate the relevance of the FC data for deducing the MIC of antibiotics, we used ampicillin as a model antibiotic and, three *E. coli* clinical isolates, two with an MIC of 4 mg/L and one with an MIC of 64 mg/L. We treated log-phase cultures of these strains with various concentrations of ampicillin: 0, 1, 2, 4, 8, 16, 32, 64, and 128 mg/L for 4 h. Aliquots of cells were harvested every hour, stained with AFH and TO-PRO®-3 and analyzed with FC (Figure 3 and Table S1).

For all strains, less fluorescent cells were stained with AFH when they were treated with ampicillin below the 0.5 MIC. This shows that detection of carbonylation of cellular macromolecules by AFH is powerful method, which allows revealing the physiological changes caused by antibiotics before complete growth inhibition. Two percent of the cells and 30% were stained when the strains with an MIC of 4 mg/L were treated with 1 and 2 mg/L of ampicillin at least 2 h, and less than 5% were stained when the strain with an MIC of 64 mg/L was treated with 1, 4, 8, and 16 mg/L of ampicillin, while 30% of the cells were stained when it was treated with 32 mg/L of ampicillin. For all strains, ≥ 60% of cells were stained with AFH when they were treated with ampicillin at a level ≥ the MIC at least 2 h. When comparing the two strains having an MIC of 4 mg/L, the percentage of cells stained with AFH was different after treatment with 4 mg/L of ampicillin. This discrepancy could indicate that the MIC of the two strains is slightly different.

For all strains, except the one having an MIC of 64 mg/L MIC treated with 32 mg/L of ampicillin, few cells were stained with TO-PRO®-3 when they were treated with ampicillin at a level < 0.5 × MIC (Figure 3). Interestingly, the percentage of fluorescent cells from the strain having an MIC of 64 mg/L, when treated with 32 mg/L of ampicillin, reached 80% after 1 h, followed by a constant decrease to 30% at 4 h. This suggests that the membrane permeabilization allowing TO-PRO®-3 to enter the cells and interact with DNA is reversible. Cells probably recovered over time through the activity of the β-lactamase expressed by this strain. For all strains, ≥ 70% of cells were stained with TO-PRO®-3 when they were treated with ampicillin at a level ≥ the MIC. For AFH and TO-PRO®-3, the percentage of stained cells was 5–20% lower when samples were treated with the highest concentrations of ampicillin than when they were treated with the concentration just above the MIC (Figure 3). This could have resulted from an increase in cell lysis at the high concentrations of ampicillin.

Each tested strain showed a particular forward scattering profile (FSC). For the ATCC25922 strain, with an MIC of 4 mg/L, the FSC ratio (treated/untreated cells) increased with the concentration of ampicillin and with incubation time, except for the samples treated with 8 mg/L of ampicillin. At this concentration, the FSC ratio increased two-fold after 1 h of incubation and then decreased. The FSC ratio of the cells treated at their MIC was ~4. For the 92030096 strain, with an MIC of 4 mg/L, the increases in the FSC ratio were higher after treatment with 2 and 4 mg/L of ampicillin than they were after treatment with the other concentrations (1, 8, 16, 32, 64, and 128 mg/L). For the 8812112 strain, with an MIC of 64 mg/L, the FSC ratios of the cells were higher after treatment with 32 and 64 mg/L of ampicillin than after treatment with 128 mg/L of ampicillin. This result was due to the presence of several subpopulations of cells among the treated cells that were larger or smaller than the untreated cells.

An analysis of the FSC/SSC areas shows that for all strains, the areas increased when the cells were treated with a concentration of ampicillin higher than the 0.5 MIC. For the 8812112 strain, with an MIC of 64 mg/L, the FSC/SSC area increased also after treatments with 0.25 MIC.

Finally, we examined the correlation between the MIC of ampicillin for the 3 strains, the ampicillin treatments and the FC measurements. To accomplish this, an X_[MIC]_ value was attributed to each experiment, i.e., one strain treated with one concentration of ampicillin over 4 h. X_[MIC]_ values were determined from the MIC of the strain and from the concentration of ampicillin used in the experiment. If for a given strain the concentration of ampicillin used in the experiment was ≥ the MIC, then X_[MIC]_ was 1. If the concentration of ampicillin used in the experiment was below the MIC, then X_[MIC]_ was —1. Four Y_[FC]_ values were calculated from the FC results obtained by measuring the following variables: the percentage of cells stained with (i) AFH or (ii) TO-PRO®-3, the change in (iii) FSC or the change in (iv) FSC/SSC area following the antibiotic treatment for each experiment. The FC results obtained were analyzed in Two ways. First, to evaluate how the MIC and the concentration of ampicillin used affected the FC results over the duration of the experiment, we calculated the areas under the curves (AUCs) of the FC results vs. the time of incubation with ampicillin (Figure 3). For each FC parameter, the coefficient of correlation (*R*^2^) between the X_[MIC]_ and Y_[FC]_ AUC was calculated for all experiments (Table 2A). Second, to evaluate how the correlations between AFH and TO-PRO®-3 staining, FSC, FSC/SSC area and MIC change over time, we calculated *R*^2^-values between all X_[MIC]_ and Y_[FC]_ comparisons at each time point for all experiments (Table 2B). Both analyses showed strong correlations between MIC values and AFH staining, TO-PRO^®;^-3 staining, and FSC/SSC area. The highest correlations were observed with AFH staining, i.e., *R*^2^-values were 0.83 for the AUCs (Table 2A) and were up to 0.85 after 2 h of incubation (Table 2B). The correlations between the MIC values and FSC were the lowest, i.e., 0.18 for the AUCs (Table 2A) and 0.39 after 1 h of incubation, after which, the correlations decreased further (Table 2A).

**Table 2:** **Correlations between MIC values, dye staining, and light scattering signals for the ampicillin-treated *E. coli* strain**.

**A**				
**AUC**				**1 × MIC *R*^2^**
AFH				0.83
TO-PRO®-3				0.75
Area FSC/SSC				0.65
FSC				0.18
**B**				
**Time (h)**	**AFH ***R***^2^**	**TO-PRO®-3 ***R***^2^**	**FSC/SSC area ***R***^2^**	**FSC ***R***^2^**
1	0.75	0.66	0.51	0.39
2	0.85	0.72	0.66	0.25
3	0.85	0.81	0.68	0.25
4	0.8	0.76	0.54	0.25

*To check if the MICs obtained using the standard microdilution assay corresponds to a threshold values impacting the FC measurements, each experiment was described by 2 variables: X_[MIC]_ and Y_[FC]_. X_[MIC]_ = 1 if the concentration of ampicillin used in the experiment ([Amp]) was ≥ 1 × MIC and X_[MIC]_ = −1 if [Amp] was < 1 × MIC. Y_[FC]_ corresponds to the FC data collected for each parameter for each experiment. **(A)** Y_[FC]_ corresponds to the areas under the curves (AUCs) of the FC results relative to the time of incubation. **(B)** Y_[FC]_ corresponds to the FC values obtained at each hour of incubation. The correlation coefficients (R^2^) between X_[MIC]_ and Y_[FC]_ for each measured parameter (% of cells stained with AFH or TO-PRO®-3, ratios of FSC and the ratios FSC/SSC areas) were calculated for all experiments. Two-tailed P < 0.0001*.

Thus, these results show that there is a strong correlation between the MIC of a strain and the FC results obtained after ampicillin treatment. Furthermore, it is possible to evaluate the MIC of a strain based on flow cytometry data obtained using the dyes AFH, TO-PRO®-3 and the light scattering data represented by the FSC/SSC area after 1 h of treatment with a range of ampicillin concentrations.

### Detection of the susceptibility of the *Pseudomonas aeruginosa* to imipenem

We decided to evaluate if the FC-AST can be used for bacterial species other than *E. coli*. For this, we tested the susceptibility of *P. aeruginosa* strains to imipenem. This choice was guided by the fact that high levels of antibiotic resistance makes *P. aeruginosa* one of the most important nosocomial pathogens (Zilberberg et al., 2010). Imipenem is frequently prescribed as a first-line therapy. However, 15 to 20% of *P. aeruginosa* cells are or become resistant during treatment with this antibiotic (Zilberberg et al., 2010). We first tested the susceptibility of log-phase cultures of two *P. aeruginosa* strains (one sensitive, one resistant) after treatment with imipenem at 2 mg/L, which corresponds to the MIC of the sensitive strain. Similar to our previous observations with *E. coli* (Figure 2), the light scattering profile of treated *P. aeruginosa* cells became dispersed, revealing a heterogeneous cell population (Figure S5A). A large proportion of the treated cells had a higher SSC and FSC value than the untreated cells. Less than 5% of the treated cells had a level of far-red autofluorescence greater than that of the untreated cells (Figure S5A). Approximately 15% of the treated cells had green autofluorescence intensity levels that were 10-fold higher than those of the untreated cells (Figure S5A).

Several studies have reported an intrinsic permeability of this bacterium to the viability dyes, which makes FC viability testing difficult (Gauthier et al., 2002; Huang et al., 2015). Indeed, we observed that the fluorescent signals of the untreated *P. aeruginosa* cells shifted slightly after staining with AFH and substantially after staining with TO-PRO®-3 when compared with the fluorescent signals of the unstained samples (Figure S5B). However, these background fluorescences did not impair the measurements of imipenem susceptibility because a much greater increase in fluorescence was observed for the antibiotic-treated populations relative to that of the untreated control populations.

As for the *E. coli* strains, a significant increase of the MFI of the cell population treated with the MIC of imipenem and stained with all dyes was observed (Figure S5C). The percentage of cells that were fluorescent due to the antibiotic treatment was calculated by using the fluorescence of the untreated, stained cells as a baseline. A large increase in the percentage of fluorescent cells stained with AFH and TO-PRO®-3 was observed after treatment with imipenem, which is similar to our data for *E. coli* (Figure S5A). However, for DiBAC4(3) staining, only 20% of the *P. aeruginosa* cells were stained after 1 and 2 h of incubation with imipenem (Figure S5A), which is much less than the 70% staining of treated *E. coli* cells (Figure 2).

**Figure 4:**
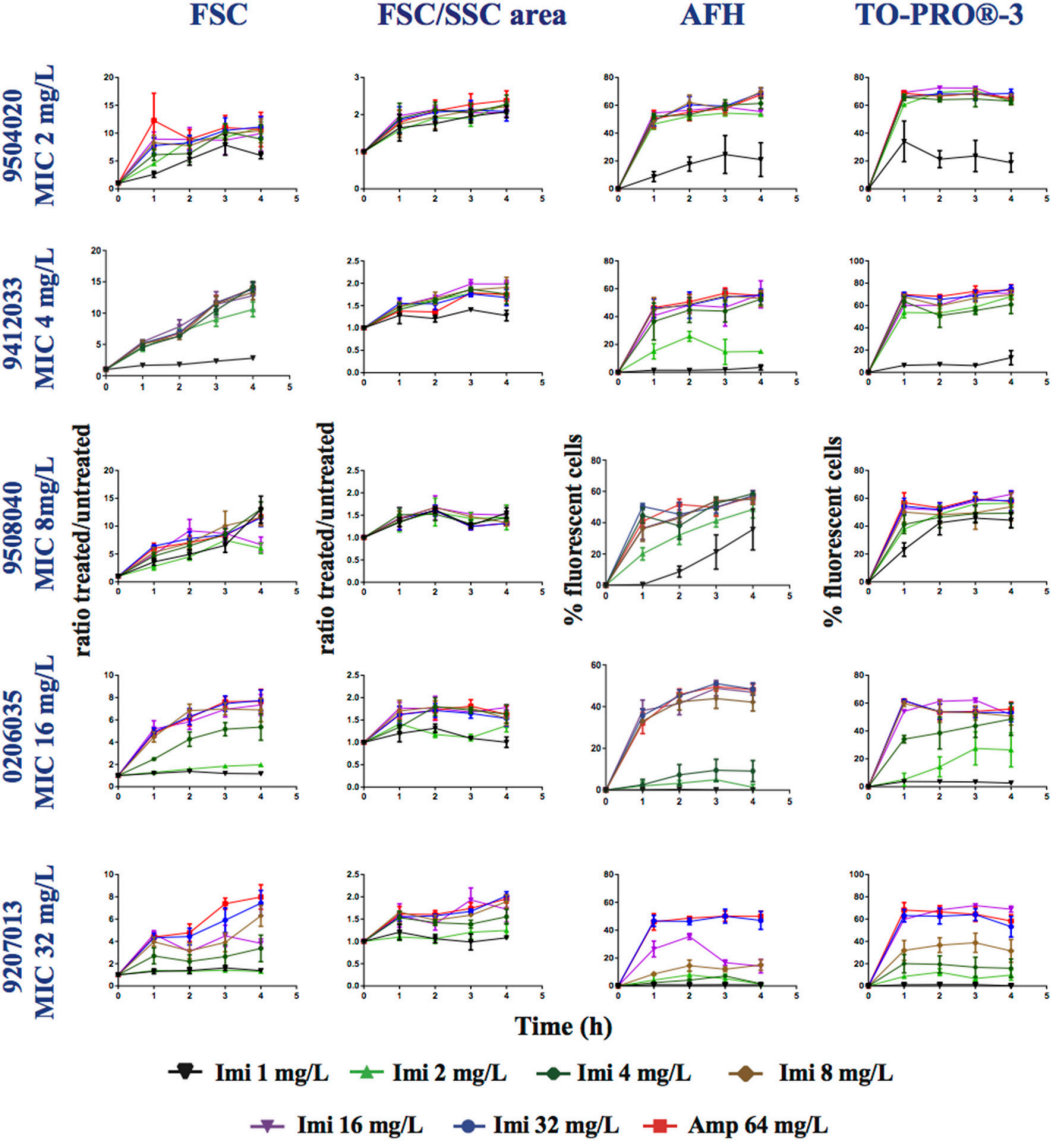
**Monitoring alterations of the *P. aeruginosa* strains treated with a range of imipenem concentrations via flow cytometry**. *P. aeruginosa* strains having different MICs (see Table 1) for imipenem were treated with a range of concentrations of imipenem (1, 2, 4, 8, 16, 32, or 64 mg/L). This figure shows the ratios of the mean FSC intensity of the imipenem-treated samples/the mean FSC intensity of the untreated samples, the ratios of the FSC/SSC area of the imipenem-treated samples/the FSC/SSC area of the untreated samples, and the percentage of fluorescent cells stained with AFH and TO-PRO®-3, all relative to incubation time. Each value represents the mean value obtained from four independent experiments. The error bars correspond to the standard error of the mean.

Next, we analyzed nine strains of *P. aeruginosa* displaying MICs for imipenem that ranged between 1 and 32 mg/L (Table 1). Each strain was grown in LB supplemented with (0, 1, 2, 4, 8, 16, 32, or 64 mg/L of imipenem) for 4 h, stained with AFH and TO-PRO®-3, and analyzed with FC (Figure 4 and Table S2). After 1 h of incubation with concentrations of imipenem < 0.5 × MIC, 1–20% of cells were stained with AFH and 1–40% with TO-PRO®-3. For some strains, the percentage of stained cells increased with the time of incubation. After 1 h of treatment with ≥ 0.5 × MIC of imipenem, the percentage of fluorescent cells stained with AFH and TO-PRO®-3 reached a plateau greater than 40 and 50%, respectively, for all tested strains.

The FSC light scattering profiles of the *P. aeruginosa* strains were strain dependent (Figure 4). This was also observed for the *E. coli* strains (Figure 2). However, the calculation of the FSC/SSC areas was less informative for *P. aeruginosa* than it was for *E. coli* because changes in FSC and SSC occurred even when the cells were treated with imipenem concentrations below 0.25 × MIC.

Finally, we calculated correlations between the MIC of imipenem for different strains of *P. aeruginosa* and the values analyzed via FC (i.e., AFH and TO-PRO®-3 staining result, FSC profiles and the FSC/SSC areas) (Table 3) as we did for *E. coli* (Table 2). The *R*^2^-values (two-tailed *P* < 0.0001) were 0.7, 0.53, 0.28, and 0.34 for AFH, TO-PRO®-3, FSC, and the FSC/SSC area, respectively. As most of strains showed a high increase of fluorescent cells even at 0.5 × MIC of imipenem, we also calculated the X_[MIC]_ variables by replacing the MIC values with the 0.5 × MIC values (Table 3A). After doing so, the *R*^2^-values were 0.80, 0.75, 0.45, and 0.33 for AFH, TO-PRO®-3, FSC, and the FSC/SSC area, respectively.

**Table 3:** **Correlations between MIC values, dye staining, and light scattering signals for the imipenem-treated *P. aeruginosa* strain**.

**A**				
**AUC**	**X_[MIC]1_*R*^2^**			**X_[MIC]2_*R*^2^**
sAFH	0.70			0.80
TO-PRO®-3	0.53			0.75
Area FSC/SSC	0.28			0.45
FSC	0.34			0.33
**B**					
**Time (h)**	**AFH** **X_[MIC]2_***R***^2^**	**TO-PRO**®**-3** **X_[MIC]2_***R***^2^**	**FSC/SSC area** **X_[MIC]2_***R***^2^**	**FSC** **X_[MIC]2_***R***^2^**	
1	0.82	0.8	0.51	0.4	
2	0.81	0.70	0.52	0.17	
3	0.73	0.68	0.56	0.2	
4	0.68	0.69	0.53	0.28	

*As for E. coli and ampicillin in Table 2, each experiment with P. aeruginosa and imipenem was described by 2 variables: X_[MIC]_ and Y_[FC]_, except that two X_[MIC]_ values were calculated. X_[MIC]1_ = 1 if the concentration of imipenem used in the experiment ([Imip]) was ≥ 1 × MIC and X_[MIC]2_ = 1 if the [Imip] was ≥ 0.5 × MIC. X_[MIC]1_ = −1 if the [Imip] was < 1 × MIC and X_[MIC]2_ = −1 if the [Imip] was < 0.5 × MIC. **(A)** R ^2^- values calculated between X_[MIC]1_ and Y_[FC]_ (AUC). R^2^-values calculated between X_[MIC]2_ and Y_[FC]_ (AUC). **(B)** R^2^-values calculated between the X_[MIC]2_ and Y_[FC]_ values for each hour of incubation. Two-tailed P < 0.0001*.

Calculations using the FC values at the different time points (Table 3B) showed that *R*^2^-values were maximal after 1 h of treatment: AFH = 0.82 and TO-PRO®-3 = 0.80. After this time point, *R*^2^-values decreased somewhat. *R*^2^-values were 0.73 and 0.68 for AFH and TO-PRO®-3, respectively, after 3 h of treatment. This decrease is due to the increase in stained cells in the samples treated with a concentration of imipenem below 0.5 × MIC (Figure 4). Therefore, we showed that it is possible to evaluate the MIC of the *P. aeruginosa* strains after 1 h of treatment with imipenem using FC and fluorescent dyes.

### Flow cytometers

We did not observe more than 90% of the cells stained with the tested dyes, while at least 99.9% of the cells were unable to give a CFU after antibiotic treatments (Figure S1B). This discrepancy was mostly due to the threshold of detection of the BD Accuri C6 FC system (BD Biosciences) used for the study. Preliminary experiments with the Gallios FC system (Beckman Coulter) showed that higher fluorescence intensities can be obtained than with BD Accuri C6. For the same experiment, ~10% more stained cells can be detected with the Gallios than with Accuri (data not shown). The small number of unstained cells could be dead “phantom” cells without intracellular contents, unculturable cells or persister cells.

## Discussion

In this study we assessed whether FC and fluorescent dyes can be used to rapidly and reliably diagnose the antibiotic susceptibility of the bacterial pathogens *E. coli* and *P. aeruginosa*. The first conclusion from this study is that the light scatter signals, FSC and SSC, are highly dependent on the bacterial strain used. However, light scatter signals allow for the detection of the heterogeneity of morphological changes and proteins content in populations of antibiotic-treated cells (Gauthier et al., 2002; Ambriz-Avina et al., 2014). We found that the analysis of scattering area in the dot-plots of FSC/SSC allowed for a useful quantification of antibiotic-induced morphological changes. In this study, we observed a high heterogeneity for *E. coli* and *P. aeruginosa* antibiotic-treated cells. For this reason, light scatter signals could be considered in combination with others factors to avoid underestimating the effects of some antibiotics. Cells sorting and an analysis of the morphologically different subpopulations may well provide new information regarding the modes of action of antibiotics and the mechanisms of resistance.

The second conclusion we reached is that cell staining with the fluorescent dyes are more reliable indicators for antibiotic susceptibility than are light scatter signals. We tested the potential of 3 dyes for use in FC-AST: AFH, TO-PRO®-3, and DiBAC4(3). AFH and TO-PRO®-3 are particularly suitable for a FC-AST because they have a far-red fluorescence, which does not increase significantly in unstained cells treated with the different antibiotics. However, green autofluorescence does increase in antibiotic treated cells (Renggli et al., 2013) and this can interfere with the detection of signals from dyes, such as DiBAC4(3) that produce a green fluorescence with some antibiotics and in some bacterial strains. For example, while the number of DiBAC4(3) stained cells increased significantly in populations of *E. coli* strains treated with the β-lactams and *P. aeruginosa* strains treated with imipenem, the increase was low and was difficult to distinguish from the green autofluorescence of the *E. coli* cells treated with the gentamicin and ciprofloxacin.

For all of the different combinations of sensitive bacterial strains and antibiotics analyzed, we observed a significant increase in the cells stained with AFH and TO-PRO®-3. However, the percentage of stained cells depended on the antibiotic used. When cells were treated with 1 × MIC of β-lactam, most of the cells were already stained with these two dyes after 1 h. However, a much lower percentage of the cells treated with gentamicin and ciprofloxacin was stained by these dyes. We also observed that the staining of cells treated with the MIC of gentamicin and ciprofloxacin was lower in fluorescence intensity and detectable after a longer time of treatment using the BD Accuri C6 cytometer than when it was treated with higher doses. One possible explication is that the impaired protein synthesis caused by gentamicin and the impaired DNA replication and transcription caused by ciprofloxacin result in slower membrane permeabilization and lower carbonylation of macromolecules than in cells treated with β-lactams.

The FC data show that the percentage of antibiotic-treated cells stained with TO-PRO®-3 was higher than the percentage of cells stained with the AFH (5–15%). This could be explained by the fact that antibiotics can cause membrane damage, allowing TO-PRO®-3 to enter the cells without killing them. Indeed, it has been reported that live cells with damaged membranes that are permeable to DNA-binding dyes can recover (Frankenberg-Schwager et al., 1975; Ruiz and Silhavy, 2005). This could explain why we found similarly high numbers of sensitive *E. coli* cells of the ATCC25922 strain stained with TO-PRO®-3 following treatments with ampicillin, cefoxitin or imipenem even though the CFU analysis showed that ampicillin killed more cells (99%) than cefoxitin and imipenem (85 and 95%, respectively), after 4 h of treatment. Staining with AFH showed a better correlation with the CFU count because it was higher with ampicillin (80% of cells stained) than with cefoxitin or imipenem treatment (60% of cells stained). Transient membrane permeabilization and cell recovery can also explain the fact that most of the cells of from an *E. coli* resistant strain treated with a sub-lethal concentration of ampicillin were transiently stained with TO-PRO®-3. Therefore, the third conclusion that we can draw from our results is that AFH is more suitable for FC-AST than TO-PRO®-3 and probably than all other DNA-interacting dyes. This observation supports our hypothesis that carbonylation, which has not previously been tested for use in FC-AST studies, is a good indicator for detecting the susceptibility of bacteria to antibiotics.

Finally, we evaluated the possibility that FC-AST can be used to determine the MIC of different bacterial strains. For this we compared FC-AST data obtained from bacterial strains having different MICs. We tested ampicillin for *E. coli* and imipenem for *P. aeruginosa*, using AFH and TO-PRO®-3 as the dyes. For *E. coli*, sub-MIC ampicillin treatment (< 0.5 MIC) resulted in a small number of cells being stained with AFH and TO-PRO®-3, while treatments at levels ≥ the MIC increased the number of stained cells by >50% and increased the dispersion of the light scatter signals (area of the density dot plots of FSC/SSC). The correlation between MIC and staining with each dye was strong: the *R*^2^ was 0.75 after 1 h of incubation and 0.85 after 2 h of incubation. These results indicate that the inhibition of growth is associated with the rapid killing of most of the cells.

Determining the MIC for the imipenem-treated *P. aeruginosa* strains was less straightforward because a substantial number of cells were stained with AFH and even more with TO-PRO®-3 at levels of imipenem ≤ 0.25 × MIC. This could be due to the previously reported artifactual enhancement of membrane permeability due to the interaction of the dye with the outer membrane (Gauthier et al., 2002). This could also be due to a growth bistability of the cells as defined by Deris et al. (2013), who showed that isogenic *E. coli* cells can coexist in growing and non-growing states over a broad range of antibiotic concentrations below the MIC. We observed that the number of CFUs in the population of *P. aeruginosa* cells treated with ≤ 0.25 × MIC was slighty lower than those of the untreated population (Figure S5). However, despite these known difficulties in analyzing *P. aeruginosa* via FC, the correlation between MIC and AFH staining was high, i.e., *R*^2^ = 0.70 after 1 h of treatment. We also observed that the increase in the percentage of cells stained with AFH was well correlated with the treatment of *P. aeruginosa* cells with imipenem at 0.5 × MIC (*R*^2^ = 0.80). A larger variety of strains should be used to confirm that 0.5 × MIC is the threshold that can be used to interpret FC-AST results for *P. aeruginosa*.

Our final conclusion is that FC-AST based on AFH staining could be used to determine the MIC of different bacterial strains. AFH-based FC-AST could even allow for more accurate measurements of MIC to be obtained as we observed noticeable differences in the FC results for *E. coli* and *P. aeruginosa* strains that have previously been shown to have the same MIC based on the standard microdilution assay. Our preliminary data with *Staphylococcus aureus* suggest that FC-AST based on AFH staining could be also used to evaluate antibiotic susceptibility of the Gram-positive bacteria (Figures S8, S9; Supplementary results 1). Therefore, it is possible that growth-based MICs could be substituted with a new FC-based standard, e.g., the minimum concentration of antibiotic needed to obtain a large increase in the number of cells stained with a dye after 2 h of incubation. The standardization of multi parametric analyses combining dye staining with morphological data obtained via light scattering signals and the use of ImageStream technology could allow for the development of a fast and reliable AST procedure. Finally, the utilization of new fluorescent reporters allowing the detection of metabolic changes that occur as a result of treatment with antibiotics could further improve the reliability of FC-AST.

## Author contributions

CS: Substantial contributions to the conception of the work; drafting of the work; analysis and interpretation of data. SC, JO, and JS: Acquisition and analysis of data for the work. CF: Design of the work. SO: Design of the work; Final approval of the version to be published. MR: Final approval of the version to be published. IM: Drafting the work, revising it critically for important intellectual content; Final approval of the version to be published.

### Conflict of interest statement

The authors declare that the research was conducted in the absence of any commercial or financial relationships that could be construed as a potential conflict of interest.
