# Supplementary material for: Antibiotic Susceptibility Testing of the Gram-Negative Bacteria Based on Flow Cytometry
Source: Front Microbiol. 2016 Jul 26;7:1121. doi: 10.3389/fmicb.2016.01121 (PMC4960253; doi:10.3389/fmicb.2016.01121)
Supplement: Supplementary file 1 [file Presentation1.pdf]

Supplemental Material

Supplemental Figure S1

A

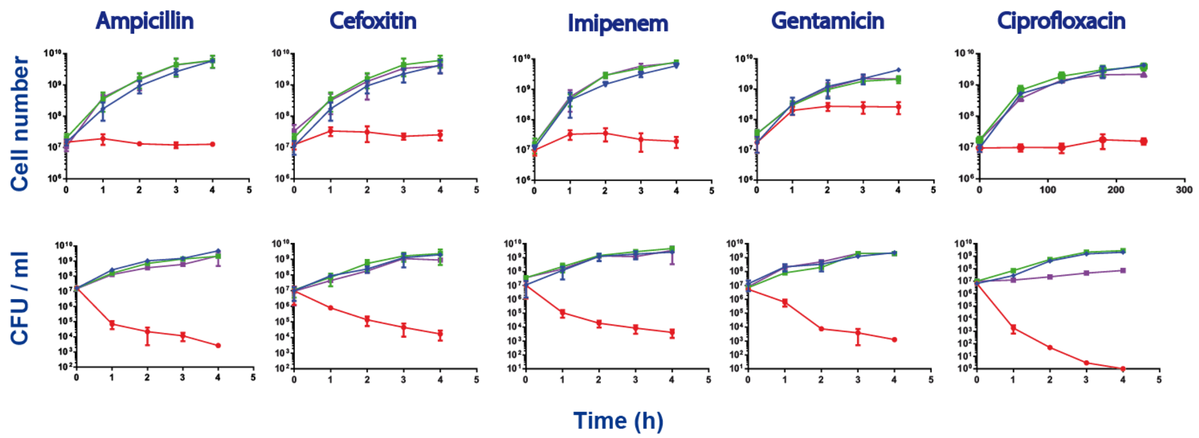

B

% of cells surviving to antibiotic treatment

Time (h)	Amp	Cefox	Imi	Gent	Cip
1	0.30	11.8	1.43	8.14	0.01
2	0.13	4.2	0.23	0.09	8.50E-04
3	0.06	1.0	0.11	0.06	4.45E-05
4	0.01	0.15	0.05	0.02	1.06E-05

Number of cells and the colony forming units in populations of *E. coli* strains treated with different antibiotics.

Time course study of the *E. coli* ATCC25922 strain sensitive to all studied antibiotics and of the resistant strains treated, or not, with ampicillin, cefoxitin, imipenem, gentamicin and ciprofloxacin. This figure shows, for each antibiotic treatment, (A) the number of cells per ml of culture and the colony forming units (CFU) per ml of culture relative to incubation time (-●-: sensitive antibiotic-treated strain, -■-: sensitive untreated strain, -■-: antibiotic-treated resistant strain, -■-: untreated resistant strain) and (B) the percentage of surviving cells was calculated as number of CFUs at each time point during the incubation with the antibiotic / number of cells at time 0.

Supplemental Figure S2

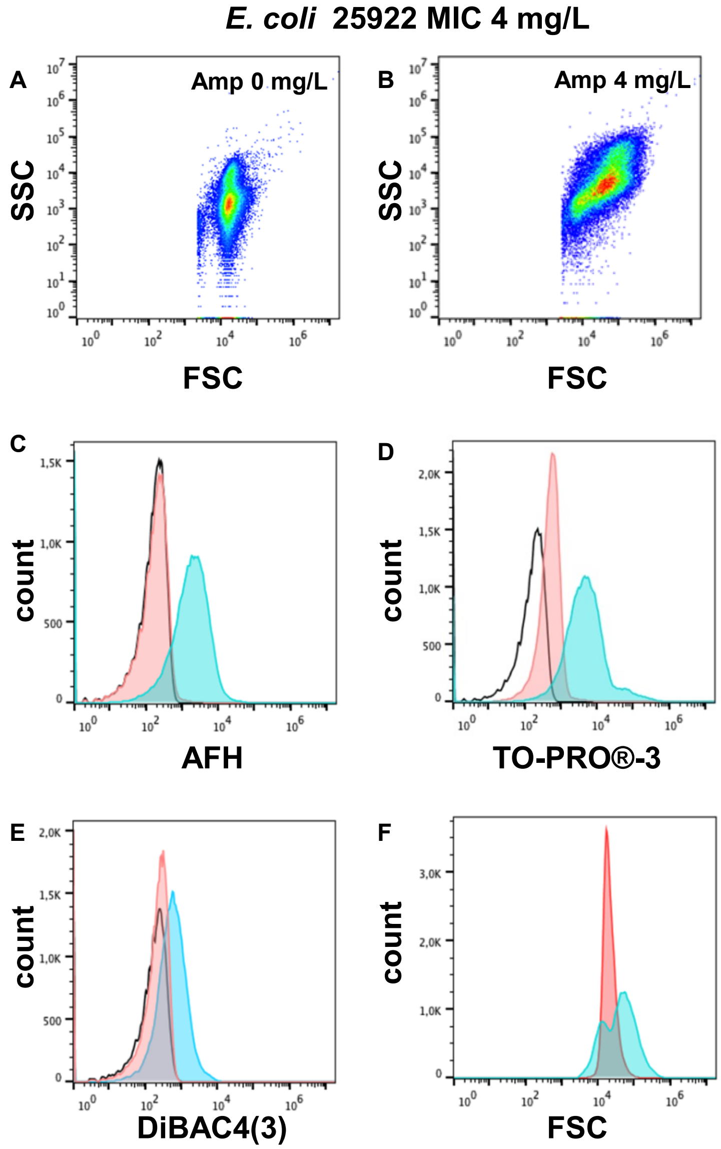

Flow cytometric analysis of the ampicillin-treated *E. coli* strain.

Light scattering profiles FSC versus SSC of the *E. coli* 25922 strain incubated for 1 hour without (A) or with ampicillin at 1 × MIC (B). Overlay histograms of the fluorescence intensity of the cells stained with (C) AFH, (D) TO-PRO®-3, (E) DiBAC4(3), (F) Overlay histogram of FSC intensities. red histogram: untreated cells, blue histogram: cells treated with ampicillin at 1 × MIC, and the black histogram represents the control (i.e., untreated, unstained cells).

Supplemental Figure S3

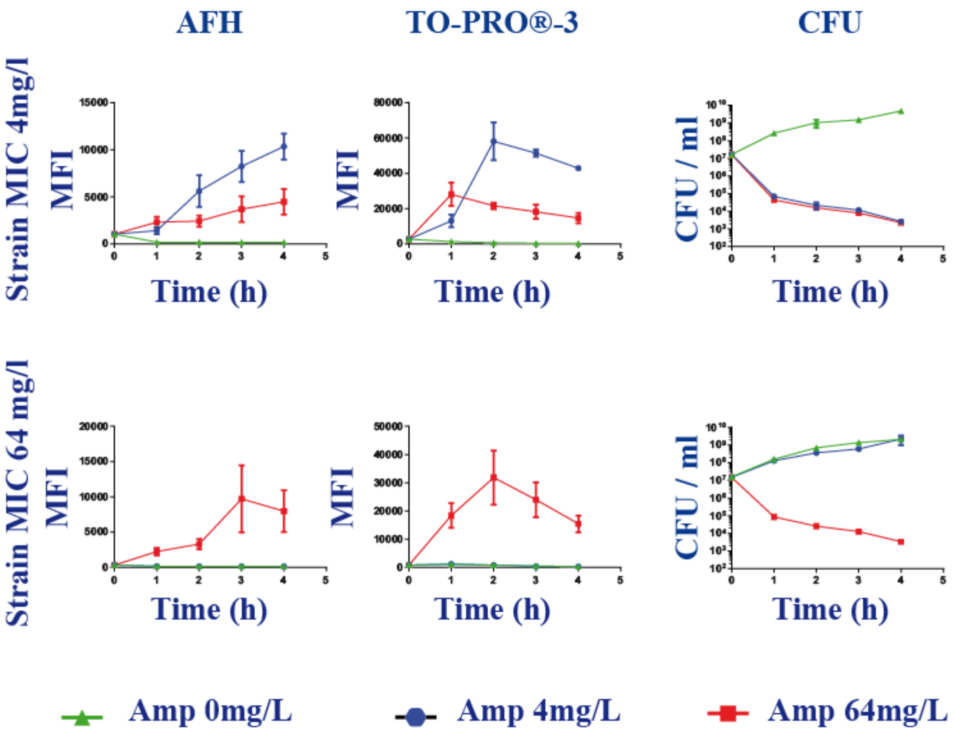

Growth curves and mean AFH and TO-PRO®-3 fluorescence intensities of *E. coli* cells treated with ampicillin.

This figure shows: the curves of the mean fluorescent intensity of the ampicillin treated (4 mg/L and 64 mg/L), and untreated, sensitive *E. coli* (ATCC 25922, MIC 4 mg/L) cells and the resistant *E. coli* (8812112, MIC 64 mg/L) cells stained with AFH or TO-PRO®-3, versus time of incubation; and the corresponding growth curves (CFU per ml versus time of incubation).

Supplemental Figure S4

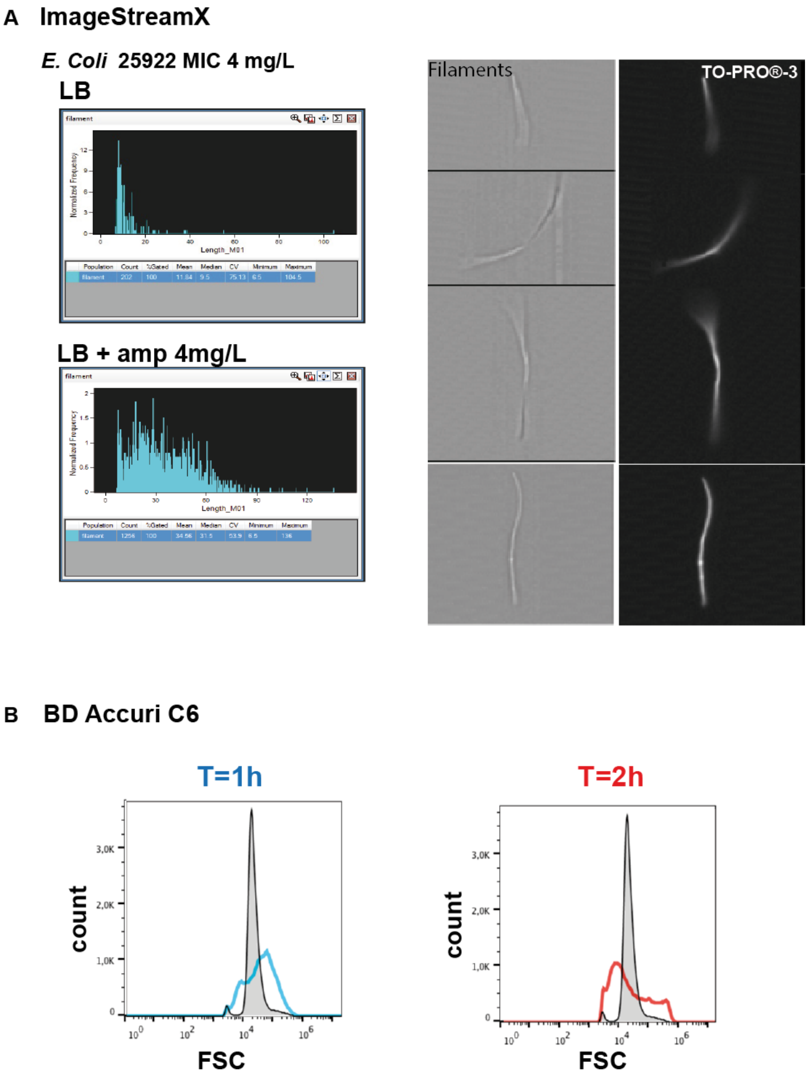

Phenotypic heterogeneity of cells in the ampicillin-treated *E. coli* population.

(A) *E. coli* ATCC2592 strain samples treated with or without ampicillin at 1 × MIC were analyzed with the ImageStream imaging flow cytometer system (Merk Millipore, Darmstadt, Germany) after 1 hour of incubation. Left: histograms of the size of the cells (gated on cells longer than 5 µm); right: images of filaments stained by TO-PRO®-3. (B) BD Accuri C6 histograms of the light scattering FSC intensities of *E. coli* ATCC 25922 strain samples treated with ampicillin at 1× MIC for 1 (blue line) or 2 (red line) hours. The untreated sample is represented in black. Within the treated cell populations, cells smaller and larger than the cells in the untreated samples were visible. The population of smaller cells, which increased with the duration of the incubation, could reflect cell lysis and shrinking.

Supplemental Figure S5

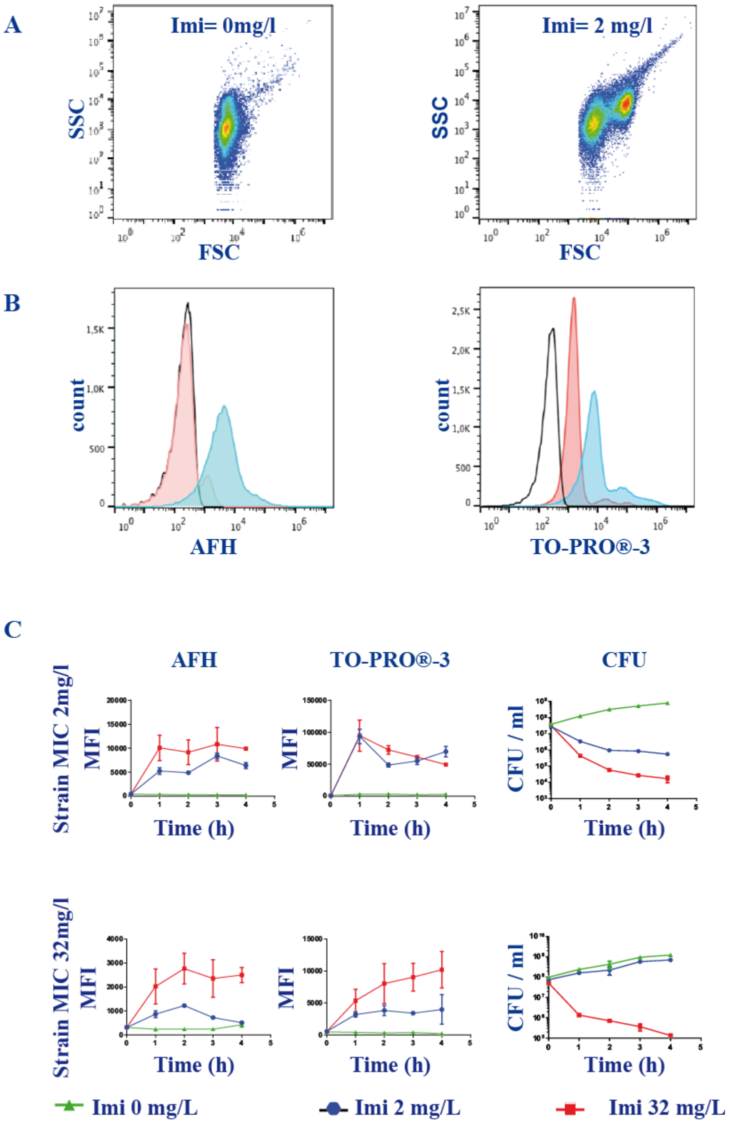

Flow cytometric analysis of the imipenem-treated *P. aeruginosa* strains.

(A and B) Flow cytometry data obtained from the sensitive *P. aeruginosa* 9504020 strain (MIC = 2 mg/L) (strain S). (A) Light scattering profiles of FSC versus SSC after 1 hour of incubation without or with imipenem (2 mg/L). (B) Overlay histograms of the fluorescence intensity of the cells stained with AFH or TO-PRO®-3. The blue histogram represents the cells treated with imipenem at 1 × MIC and stained with dye, the red histogram represents the untreated cells stained with dye, and the black histogram represents the control (i.e., untreated, unstained cells). (C) Curves of the mean fluorescent intensity of the imipenem (2 mg/L and 32 mg/L) treated, and untreated, sensitive *P. aeruginosa* (9504020, MIC 2 mg/L) and resistant *P. aeruginosa* (9207013, MIC 32mg/L) strain cells stained with AFH or TO-PRO®-3, versus time of incubation; and the corresponding growth curves (CFU per ml and number of cells per ml versus time of incubation).

Supplemental Figure S6

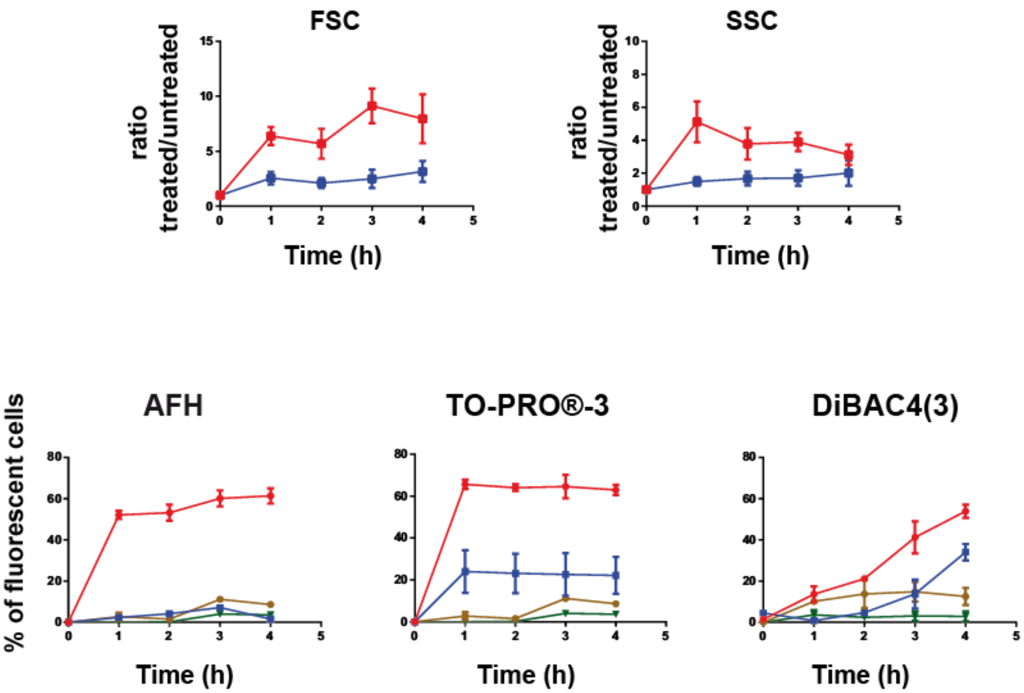

Monitoring sensitive and resistant *P. aeruginosa* cells treated with imipenem. Time course study of the sensitive and resistant *P. aeruginosa* cells treated, or not with imipenem. The ratios of the mean FSC intensity of the antibiotic-treated sample / mean FSC intensity of the untreated sample and the ratios of the mean SSC intensity of the treated sample / mean SSC intensity of the untreated sample, both relative to incubation time of the S strain and the 9207013 strain resistant to imipenem (MIC = 32 mg/L) (strain R), treated, or not, with imipenem at 1 × MIC. The percentage of fluorescent cells stained with AFH, TO-PRO®-3 or DiBAC4(3) from the strain S and the strain R treated or untreated with imipenem at 1 × MIC relative to time. -●-: antibiotic-treated strain S, -■-: antibiotic-treated strain R, -◆-: autofluorescence of antibiotic-treated strain S, -▼-: autofluorescence of the antibiotic-treated strain R (Ex 640 nm, Em 675 nm ± 25 nm for AFH and TO-PRO®-3; Ex 488 nm, Em 530 nm ± 30 nm for DiBAC4(3))

Supplemental Figure S7

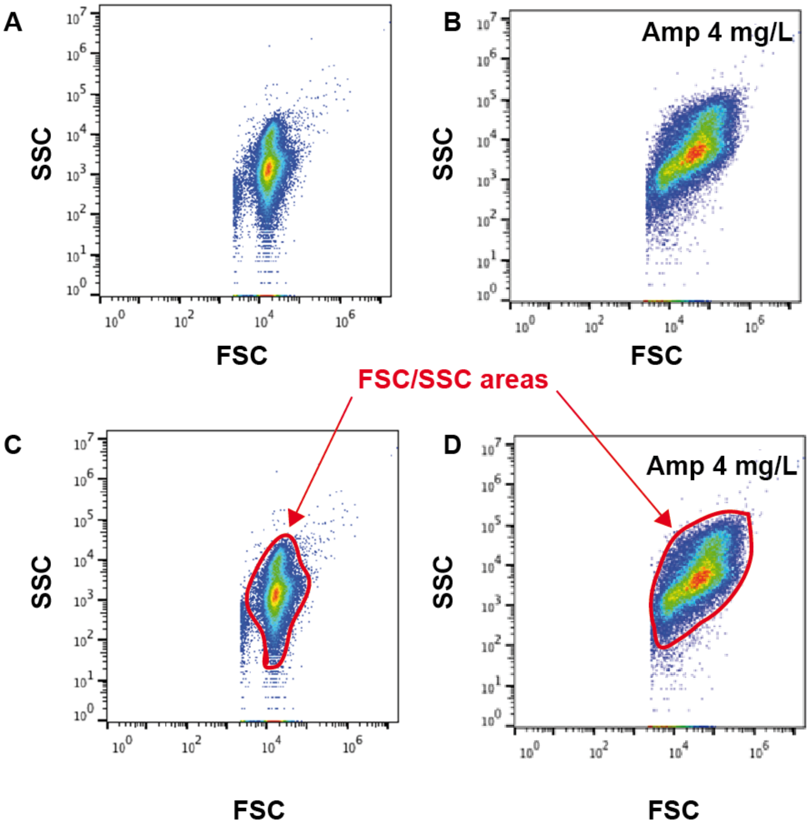

Measurement of the areas of pseudocolor as a density-plot of FSC/SSC (FSC/SSC areas).

E. coli ATCC2592 strain treated (B, D) or not (A, C) with ampicillin at 1 × MIC. The FSC/SSC areas were measured based on the pseudocolor density-plot of FSC/SSC using the “polygon selection” tool of the ImageJ 1.48v software (areas are underlined in red (C, D).

Supplementary Table S1

AUC AFH

		ampicillin treatment							
**strains**	**MIC**	1 mg/L	2 mg/L	4 mg/L	8 mg/L	16 mg/L	32 mg/L	64 mg/L	128 mg/L
25922	4 mg/L	6.14	152.7	249.7	241.8	185.6	192.7	175.9	188.2
92030096	4 mg/L	3.833	116	153.3	242.3	224.3	217.5	177.8	213.5
8812112	64 mg/L	1.055	3.925	1.148	1.428	15.7	56.83	184.9	248.6

AUC TO-PRO®-3

		ampicillin treatment							
**strains**	**MIC**	1 mg/L	2 mg/L	4 mg/L	8 mg/L	16 mg/L	32 mg/L	64 mg/L	128 mg/L
25922	4 mg/L	26.82	234.7	295.3	292.4	282.8	277.1	288.9	284.8
92030096	4 mg/L	28.15	231	296.1	303.3	270.6	266.5	286.4	257.1
8812112	64 mg/L	1.183	0.3875	1.61	1.743	104.7	197.5	322.3	291.1

AUC FSC treated /untreated

		ampicillin treatment							
**strains**	**MIC**	1 mg/L	2 mg/L	4 mg/L	8 mg/L	16 mg/L	32 mg/L	64 mg/L	128 mg/L
25922	4 mg/L	5.751	11.5	15.39	9.205	16.66	22.69	19.51	31.7
92030096	4 mg/L	5.59	16.12	19.29	6.348	8.593	6.833	8.678	9.146
8812112	64 mg/L	4.102	4.06	4.112	4.159	7.529	11.94	22.51	6.796

AUC ratios areas FSC/SSC treated/untreated

		ampicillin treatment							
**strains**	**MIC**	1 mg/L	2 mg/L	4 mg/L	8 mg/L	16 mg/L	32 mg/L	64 mg/L	128 mg/L
25922	4 mg/L	5.105	5.68	7.535	7.617	7.499	7.537	7.801	7.233
92030096	4 mg/L	4.503	8.662	8.153	8.125	8.39	8.739	8.915	8.774
8812112	64 mg/L	3.956	4.102	4.009	3.817	6.245	7.219	7.674	8.127

***E. coli* experiments.**

The values of the areas under the curves (AUC) were calculated from the curves of the FC measurement values relative to time (4 hours) (median values of 4 experiments).

Supplementary Table S2

AUC staining AFH

		imipenem treatment						
**strains**	**MIC**	1 mg/L	2 mg/L	4 mg/L	8 mg/L	16 mg/L	32 mg/L	64 mg/L
9203182	1 mg/L	204.4	nd	205.5	207.1	nd	202.5	209.7
9405057	2 mg/L	133.3	nd	143.1	137.6	nd	146.1	130.5
9504020	2 mg/L	61.85	177.6	196.1	203.2	197.4	203.4	196.8
9412033	4 mg/L	6.83	63.15	151.4	175.6	163.7	175.9	181.5
9508040	8 mg/L	48.34	117.5	164.2	160.7	161.3	174.3	170.6
206035	16 mg/L	0.565	10.97	23.98	140.3	151.9	156.2	151.6
9207006	16 mg/L	4.637	nd	27.83	145.8	nd	150.9	157.6
1011315	16 mg/L	2.85	nd	123.1	168.1	nd	181.9	182.2
9207013	32 mg/L	2.952	18.22	14.49	42.79	85.95	166.7	169.8

AUC TO-PRO®-3

		imipenem treatment						
**strains**	**MIC**	1 mg/L	2 mg/L	4 mg/L	8 mg/L	16 mg/L	32 mg/L	64 mg/L
9203182	1 mg/L	147.1	nd	167.1	177.8	nd	174.3	177.4
9405057	2 mg/L	206.1	nd	203.1	213	nd	213.9	214.1
9504020	2 mg/L	88.53	231.6	225.8	233.8	245.8	236.5	236.4
9412033	4 mg/L	25.99	199.5	200.3	229	226.9	240.8	247.6
9508040	8 mg/L	133.5	170.8	161.3	174.8	193.6	194.4	198.5
206035	16 mg/L	12.13	60.2	140.6	192.1	203.4	195.7	197.2
9207006	16 mg/L	9.345	nd	126.1	184.9	nd	200.6	196.8
1011315	16 mg/L	4.247	nd	161.1	190.7	nd	201.4	201
9207013	32 mg/L	3.603	32.52	64.15	123.1	234.5	216.4	228.1

AUC ratios FSC treated/untreated

		imipenem treatment						
**strains**	**MIC**	1 mg/L	2 mg/L	4 mg/L	8 mg/L	16 mg/L	32 mg/L	64 mg/L
9203182	1 mg/L	54.84	nd	87.91	102.7	nd	84.78	98.44
9405057	2 mg/L	14.61	nd	17.18	17.82	nd	28.18	15.8
9504020	2 mg/L	19.3	28.51	27.69	31.72	31.99	32.6	38
9412033	4 mg/L	7.653	26.19	29.01	30.08	31.56	31.17	30.94
9508040	8 mg/L	22.06	18.29	26.7	28.61	26.63	28.92	27.73
206035	16 mg/L	4.863	6.211	15.09	22.22	22.13	23.03	22.95
9207006	16 mg/L	5.134	nd	15.17	20.26	nd	28.55	25.35
1011315	16 mg/L	4.62	nd	19.42	22.48	nd	24.1	22.78
9207013	32 mg/L	5.504	5.276	9.729	14.69	14.64	18.88	21.05

AUC ratios area FSC/SSC treated/ untreated

		imipenem treatment						
**strains**	cmi	1 mg/L	2 mg/L	4 mg/L	8 mg/L	16 mg/L	32 mg/L	64 mg/L
9203182	1 mg/L	6.902	nd	7.05	7.216	nd	6.926	7.447
9405057	2 mg/L	5.924	nd	6.103	5.778	nd	5.493	5.557
9504020	2 mg/L	6.896	7.031	7.715	7.4	7.681	7.59	7.855
9412033	4 mg/L	5.046	6.255	6.275	6.463	6.665	6.196	5.898
9508040	8 mg/L	5.504	5.584	5.562	5.73	5.877	5.427	5.427
206035	16 mg/L	4.607	4.885	6.174	6.529	6.593	6.248	6.467
9207006	16 mg/L	3.869	nd	5.973	6.834	nd	6.784	6.689
1011315	16 mg/L	4.385	nd	7.275	7.591	nd	7.695	7.59
9207013	32 mg/L	5.046	4.485	6.275	6.463	6.264	6.196	5.898

***P. aeruginosa* experiments.**

The values of the areas under the curves (AUC) were calculated from the curves of the FC measurement values relative to time (4 hours) (median values of 4 experiments). nd: not done.

Supplementary results: Detection of the susceptibility of the *Staphylococcus aureus* to ciprofloxacin.

We tested our methodological approach on a Gram-positive bacterium, *Staphylococcus aureus*. As with *P. aeruginosa*, *S. aureus,* in particular the methicillin-resistant *S. aureus* (MRSA), is a pathogen frequently involved in nosocomial infections. We chose to analyze the susceptibility of the *S. aureus* bacterium to ciprofloxacin because fluoroquinolones are among the drugs of choice used to treat MRSA infections. However, the MRSA, which are multidrug resistant strains, are frequently resistant to ciprofloxacin (Michel and Gutmann, 1997; Weber et al., 2003). Two strains were analyzed: one sensitive (strain 8311065; MIC: 0.25 mg/L) and one resistant (strain 0807063; MIC: 32 mg/L) to ciprofloxacin. For each experiment, log-phase cultures of the sensitive strain and the resistant strain were treated, or not, with ciprofloxacin and analyzed in a 4-hour time course study. The collected FC data are reported in the **Figure S8**.

Light scattering profiles FSC and SSC. We observed that the light scattering profile of the sensitive strain treated with 1 mg/L of ciprofloxacin became dispersed (**Figure S8 A**). These changes were moderate compared to the FC data obtained from *E. coli*. We calculated the FSC ratios (or SSC) between the mean values of the FSC (or SSC) intensity of the treated cells and the mean values of the FSC (or SSC) intensity of the untreated cells. The FSC and SSC ratios were approximately 2 when the cells were exposed for 2 hours to ciprofloxacin. These ratios decreased after 3 hours of incubation because a subpopulation of cells had shrunken, while other cells were larger than the untreated cells. We also calculated the ratios between of the area of the density dot plot of the FSC/SSC ratios of the treated cells and the untreated cells for each experiment. The differences observed between the ratios (FSC, SSC and FSC/SSC area) of the sensitive strain and those of the resistant strain were all significant (t-test; p value < 0.03), except for the difference between the ratios of SSC obtained at 4 hours of incubation (t-test, p value = 0.09) (**Figure S8 C**).

Autofluorescence. Exposure of cells from the 2 strains of *S. aureus* to 1 mg/L of ciprofloxacin induced a weak cell far-red (excitation at 633 nm, emission at 660/20 nm) and blue (excitation at 488 nm, emission at 530/30 nm) autofluorescence. Less than 5% of the treated cells were more fluorescent than the untreated cells. In the conditions used in these experiments, the autofluorescence due to the treatment did not interfere with the FC measurements of the fluorescent dyes (**Figure S8 D**).

Changes in cells fluorescence after staining with the AFH, TO-PRO®-3 and DiBAC$_4$. After staining with AFH and TO-PRO®-3, we found a significant increase of the fluorescence intensity of the sensitive cell population treated with the ciprofloxacin compared to the untreated cell population. After staining with DiBAC$_4$ the increase of fluorescence intensity of the cell population after treatment was low (**Figure S9**). 20% of the sensitive cells exposed to ciprofloxacin for at least 2 hours and stained with AFH were fluorescent. The percentage of fluorescents cells, which increased in a time-dependent manner, reached approximately 60% after 4 hours of incubation (**Figure S8 B and D**). After staining with TO-PRO®-3, 40% of the sensitive cells were fluorescent after 1 hour of incubation. However, after 3 hours of incubation, we observed a decrease in the percentage of cells stained with TO-PRO®-3. The fact that the number of cells stained with AFH still increased after 3 hours suggests that the decrease of cell stained with the TO-PRO®-3 was not due to the restoration of membrane impermeability but rather to a change in the DNA accessibility to the dye. Nevertheless, the differences between the FC results obtained for the sensitive and resistant strains at each time point in the incubation with

AFH and TO-PRO®-3 were significant (t-tests, p value $\leqslant$ 0.005 and p value $\leqslant$ 0.04, respectively). Double staining with AFH and TO-PRO®-3 could allow the susceptibility of *S. aureus* strains to ciprofloxacin to be efficiently detected after 1 hour of incubation with the antibiotic.

As for *E. coli* treated with the ciprofloxacin, the staining of the sensitive *S. aureus* cells with $DiBAC_4(3)$ was weak. Fifteen to 20% of the cells were stained with $DiBAC_4(3)$ after 3 hours of incubation. The difference between the staining of sensitive and resistant cells with $DiBAC_4(3)$ was significant only after 3 hours of incubation (t-test p value $\leqslant$ 0.01).

Taken together, our data showed that it is possible to assess the susceptibility of a sensitive strain of *S. aureus* to ciprofloxacin using FC analysis based on AFH, TO-PRO®-3 and the light scattering data.

REFERENCES

Michel, M., and Gutmann, L. (1997). Methicillin-resistant Staphylococcus aureus and vancomycin-resistant enterococci: therapeutic realities and possibilities. *Lancet* 349**,** 1901-1906.

Weber, S.G., Gold, H.S., Hooper, D.C., Karchmer, A.W., and Carmeli, Y. (2003). Fluoroquinolones and the risk for methicillin-resistant Staphylococcus aureus in hospitalized patients. *Emerg Infect Dis* 9**,** 1415-1422. doi: 10.3201/eid0911.030284.

Supplemental Figure S8

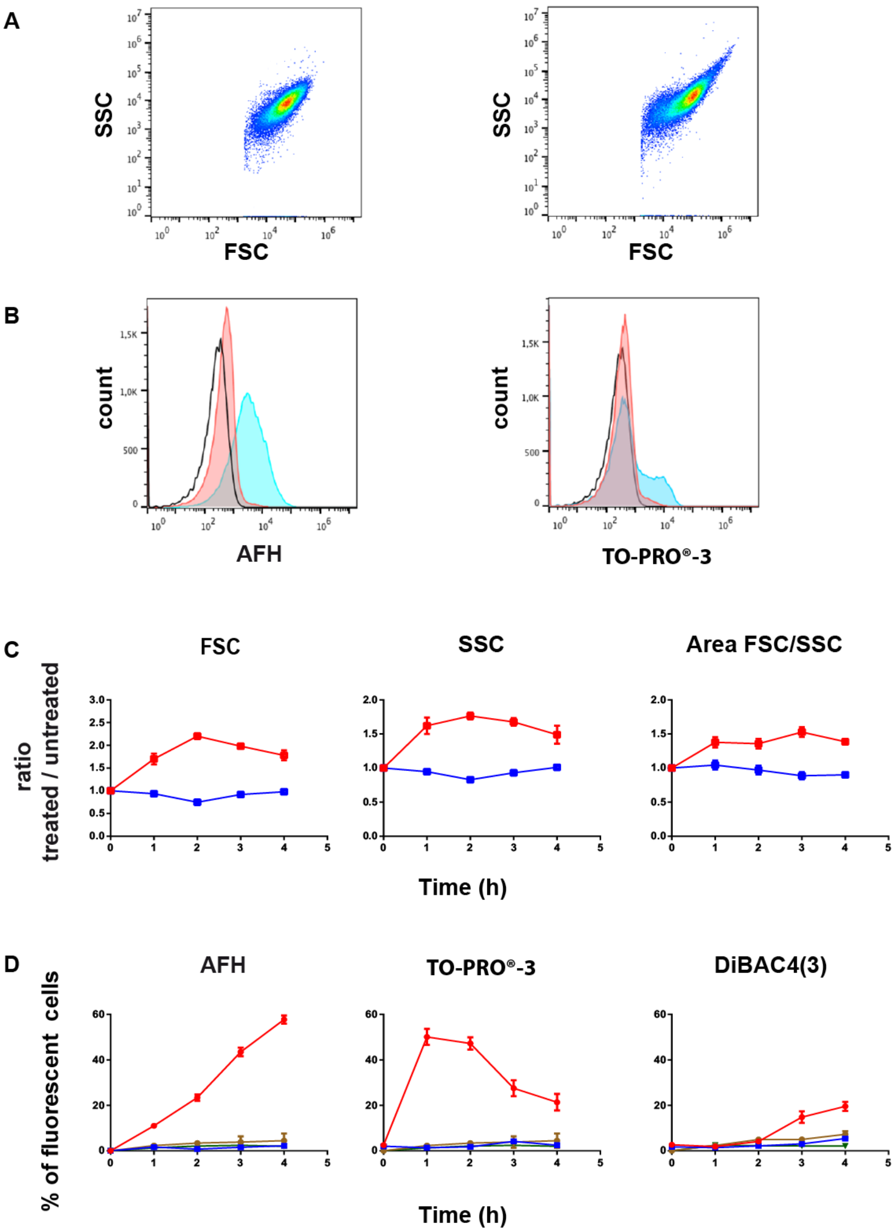

Analysis of the susceptibility of the *Staphylococcus aureus* to ciprofloxacin.

(A) Light scattering profiles for FSC versus SSC of cells from the strain 8311065 (MIC = 0.25 mg/L) after 4 hours of incubation without (left) or with 1 mg/L of ciprofloxacin (right).

(B) Overlay histograms of the fluorescent intensities of cells stained with AFH or TO-PRO®-3. The blue histogram represents the cells treated with ciprofloxacin and stained with dye, the red histogram represents untreated cells stained with dye, and the black histogram is the control (untreated, unstained cells).

(C and D) Time course study of samples from the 8311065 (MIC = 0.25 mg/L) and 0807063 (MIC = 32 mg/L) strains treated, or not, with 1 mg/L of ciprofloxacin.

(C) Left and middle: the ratios of the mean FSC (or SSC) intensities between the antibiotic-treated cells and the mean FSC (or SSC) intensities of the untreated cells from the sensitive and resistant strains relative to incubation time. Right: kinetic curve of the ratios between the area of the density plots of FSC/SSC of the treated and the untreated cells from the sensitive and resistant strains relative to incubation time.

(D) The percentage of fluorescent cells stained with AFH, TO-PRO®-3 and DiBAC$_4$(3) relative to time.

-●-: sensitive antibiotic-treated strain, -■-: resistant antibiotic-treated strain, -◆-: autofluorescence of the sensitive antibiotic-treated strain, -▼-: autofluorescence of the antibiotic-treated resistant strain (Ex 640 nm, Em 675 nm ± 25 nm for AFH and TO-PRO®-3; Ex 488 nm, Em 530 nm ± 30 nm for DiBAC$_4$(3).

Supplemental Figure S9

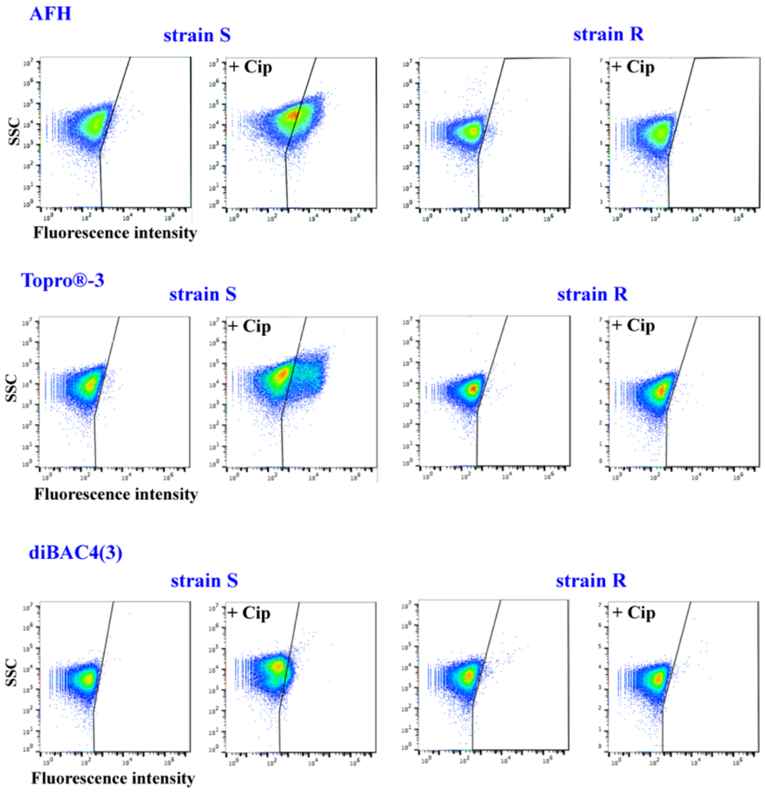

Effects of antibiotics on the light scatter SSC and fluorescence intensity distribution in *S. aureus* strains

Density-plots SSC versus fluorescence intensity of bacterial samples treated, or not, with ciprofloxacin 0.25 mg/L, during 3 hours and stained with AFH, TO-PRO®-3, and DiBAC4(3). The gate in the density-plots of the untreated samples and the density-plots of the treated samples are at the same place. Strain S: MIC = 0.25 mg/L; strain R: MIC = 32 mg/L.
